# Analysis of the etiologies of female infertility in Yunnan minority areas

**DOI:** 10.1186/s12905-021-01216-5

**Published:** 2021-03-01

**Authors:** Fen Zhang, Qing Feng, Linna Yang, Xuelian Liu, Lingyun Su, Chunyan Wang, Huimei Yao, Dawei Sun, Yun Feng

**Affiliations:** 1 Department of Obstetrics and Gynecology, The First People’s Hospital of Yunnan Province, No. 157 of Jinbi Road, Xishan District, Kunming, 650032 China; 2 Medical School of Kunming University of Science and Technology, Kunming, 650500 China; 3 Kunming Dianchi Lake Evironmental Protection Collaborative Research Center, Kunming University, Kunming, 650214 China; 4 Department of Obstetrics and Gynecology, Peking Union Medical College Hospital (Dongdan Campus), No.1 Shuaifuyuan Wangfujing, Dongcheng District, Beijing, 100730 China; 5 Department of Gynecology, Dehong Hospital of Traditional Chinese Medicine, Mangshi, 678400 China; 6 Department of Gynecology, The Second People’s Hospital of Baoshan City, Baoshan, 678000 China; 7 Department of Obstetrics and Gynecology, Cangyuan Wa Autonomous County People’s Hospital, Cangyuan, 677400 China

**Keywords:** Infertility, Etiology, Related factors, Yunnan, Minority areas

## Abstract

**Background:**

The present study aims to provide a comparative analysis of the etiologies of female infertility between Dehong, on the Yunnan Frontier, and Kunming.

**Methods:**

A retrospective study, which included 941 infertile females in Kunming who were treated in the First People’s Hospital of Yunnan Province and infertile females who were treated in the local hospital in Dehong from January 2016 to November 2018, was conducted. A comparative analysis of the etiologies of infertility in the two regions was then carried out.

**Results:**

In patients with primary infertility, ovulation disorder (15.03%) was the main cause of infertility in Kunming, and pelvic inflammatory disease (25.59%) was the main cause in Dehong. With regard to secondary infertility, although pelvic inflammatory disease was the main cause of infertility in both regions, the incidence of intrauterine adhesions in Kunming was significantly higher than in Dehong.

**Conclusions:**

The etiology of infertility showed different epidemiological characteristics depending on the region, hence individualized treatment should be given accordingly

## Background

According to criteria issued by the World Health Organization (WHO), those who are married, have a normal sex life without the use of contraception, and have lived with their partner for one year without conception are called infertile [1]. Due to influence from a given region’s environment, climate, culture, and customs, the incidence of infertility differs across regions and countries. It is reported that the incidence of infertility in developed countries is 5–8%, while in developing countries it is as high as 5.8–44.2%. This difference is also reflected in various specific countries. For example, the incidences of infertility in Iran, Britain, and China range between 3–8%, 2–26%, and 1–18%, respectively [2].

Infertility is regarded as a global problem that affects the health and economic situations of sufferers and their families [3, 4]. The inability to have a child whenever they want, can lead to a crisis situation in women because there are personal, interpersonal, social and religious expectations of having children. In developing countries, infertility causes social stigma, women diagnosed with infertility are abandoned by their husbands, become victims of spousal and family violence, are excluded from the family and society and experience a sense of failure [5].

Previous studies have revealed that differences in the etiologies of infertility are mainly due to differences in culture, socioeconomics, health care practices, policies, and environmental conditions [6]. The present study provided a comparative analysis of the etiologies of female infertility between Dehong, on the Yunnan Frontier, and Kunming. This study aimed to determine whether infertile Han females from different regions had different epidemiological characteristics.

## Methods

### Study population and sample size

This is a cross-sectional study. The random sample selection was used. From January 2016 to November 2018, 941 infertile Han females in Kunming who were treated in the First People's Hospital of Yunnan Province, and 813 infertile females who were treated in Dehong Dai and Jingpo Autonomous Prefecture Hospital, were enrolled in the study.

### Inclusion criteria

All the patients met the WHO criteria for the diagnosis of female infertility: having been sexually active for at least 12 months without using contraception without becoming pregnant. Among them, those who have never been pregnant previously fall under the category of primary infertility; those who have been pregnant in the past but who have not been pregnant for 12 consecutive months without contraception fall under the category of secondary infertility.

All patients had normal cognitive function and participated in the study voluntarily with signed informed consent.

### Exclusion criteria

Exclusion criteria for this study were infertility due to the male component, less than one year of menarche or entrance into the perimenopausal period as verified by the detection of sex hormones, malignant tumors, severe cardiovascular, cerebrovascular, hepatic, or renal disease, mental disease or cognitive impairment, unwillingness to sign informed consent, and relocation from other places to the survey sites.

### Data collection

Medical records were obtained from doctors in the two hospitals. A proforma used to extract information from the patient records. The contents of clinical data included age, educational background, nationality, residence, body mass index (BMI), marriage age, duration of disease, menstrual history, previous history of infertility, and reproductive history. The actual etiologies of infertility were obtained from the operations themselves, and the etiologies and related factors of infertility in the two regions were compared and analyzed.

### Statistic analysis

An excel spreadsheet was formulated and the collected medical history data were input by recruiting and reexamination. For those cases that required surgery, the intraoperative conditions were recorded. Double entry was adopted for data input. In case of any discrepancy in data entry, the original inquiry questionnaire and medical record data were rechecked.

SPSS 22.0 software was used for data analysis, the Pearson *χ*^2^ test was used for comparing proportions, and logistic regression analysis was adopted for the analysis of the influencing factors of infertility. P value < 0.05 was regarded as statistically significant.

## Results

### General characteristics

From the 1,754 patients analyzed in the present study, 941 were in Kunming and 813 in Dehong. There was no statistical significance in the age distribution of infertile patients between the two regions (χ^2^ = 7.42, P value = 0.192). The mean age of infertile patients in Kunming was 29.09 ± 4.81 years (range 20–41 years), and the mean age of infertile patients in Delong was 28.87 ± 5.02 years (range 19–44 years); the difference of age between the two regions was not statistically significant (P value = 0.349). The difference in the distribution of educational background, however, was statistically significant (χ^2^ = 31.87, P value < 0.001); there were more infertile patients with a high educational background (college degree or above) in Kunming than in Dehong. Statistical significance was also found in the distribution of residence in the two regions (χ^2^ = 9.71, P value = 0.021); the majority of the infertile patients (75.24%) in Kunming lived in urban areas, whereas the majority in Dehong (77.49%) lived in rural areas. Statistical analysis showed that there was a significant difference in the distribution of marriage age between the two regions (χ^2^ = 23.19, P value < 0.001); the percentage of patients with a marriage age of ≤ 25 years in the Dehong region (66.18%) was significantly higher than in Kunming (48.54%). There was no significant difference in the number of sexual encounters per week between the two regions (3.23 ± 1.81 vs. 3.12 ± 2.10, P value = 0.287). In addition, among the patients in Kunming, the number of patients who knew their most fertile days of the month was higher than in the Dehong area; the difference was statistically significant (χ^2^ = 31.52, P value < 0.001).

The majority of infertile patients in both regions had a BMI within the normal range, but the BMI of patients in Kunming was higher than in Dehong. There were more lean patients (BMI < 18.5) in Dehong than that in Kunming, and there were more overweight patients (BMI ≥ 25) in Kunming than that in Dehong (see Table 1). The difference in BMI between the two regions was statistically significant (χ^2^ = 43.32, P value < 0.001).

**Table 1:** Comparison of BMI in patients with infertility between the two regions (n = 1754)

BMI (kg/m^2^)	Kunming region (n = 941)		Dehong region (n = 813)	
	Case (n)	Percent (%)	Case (n)	Percent (%)
Underweight (< 18.5)	98	10.47	174	21.49
Normal (18.5–24.9)	664	70.51	523	64.33
Overweight (≥ 25)	179	19.02	116	14.27

*BMI* body mass index

### Comparison of medical histories

Table 2 shows that, in the medical histories of the patients in Kunming, polycystic ovarian syndrome (PCOS) accounted for the highest proportion (8.82%), followed by cervicitis (8.61%), ectopic pregnancy (7.22%), and pelvic inflammatory disease (5.95%). Medical histories for patients in Dehong showed that the proportion of pelvic inflammatory disease (15.62%) was the highest, followed by cervicitis (10.86%), ectopic pregnancy (7.01%), and vaginitis (6.27%).

**Table 2:** The distribution of past history in infertile patients between the two regions (n = 1754)

Medical histories	Kunming region (n = 941)		Dehong region (n = 813)	
	Case (n)	Percent (%)	Case (n)	Percent (%)
Pelvic inflammatory	56	5.95	127	15.62
Ectopic pregnancy	68	7.22	57	7.01
Ovarian cysts	49	5.21	40	4.92
PCOS	83	8.82	43	5.29
Adenomyosis	11	1.17	16	1.97
Endometriosis	20	2.13	39	4.80
Endometrial polyp	19	2.02	13	1.6
Intrauterine adhesions	29	3.08	15	1.85
Cervicitis	81	8.61	82	10.86
Vaginitis	24	2.55	51	6.27

*PCOS* polycystic ovary syndrome

### Analysis of pregnancy histories for patients with secondary infertility

As shown in Table 3, the pregnancy times for patients with secondary infertility differed between regions, and this difference was statistically significant. In Kunming, the number of patients with a history of more than two previous pregnancies was significantly higher than in Dehong. In Dehong, however, the majority of patients with secondary infertility had a history of only one previous pregnancy.

**Table 3:** The constituent ratio of pregnancy history in patients with secondary infertility between the two regions (n = 1089)

Groups	Kunming region (n = 655)		Dehong region (n = 434)		$\upchi$^2^	P value
	Case (n)	Percent (%)	Case (n)	Percent (%)		
G1	244	37.25	252	58.03	32.79	< 0.001
G2	202	30.84	106	24.45		
G3	112	17.10	42	9.49		
≥ G4	97	14.81	34	8.03		

G, generation; $\upchi$^2^, Chi-square

### Analysis of factors influencing infertility in patients

Logistic regression models of the factors influencing infertility were established in both locations. Based on the data from infertile patients in Dehong, the regression equation was used for the comparison between the Kunming and non-Kunming (Dehong) areas. Then, based on the data of infertile patients in Kunming, the regression equation was used for the comparison between Dehong and non-Dehong (Kunming) areas. The results were showed in Table 4. In the regression equation, marriage age, BMI, menstrual flow are the quantitative data; Menstrual regularity and pelvic inflammatory are the categorical data.

**Table 4:** Logistic analysis of multiple factors between the two regions (n = 1754)

Index	B	S.E	Wald	df	sig	OR	95% CI of OR
Kunming region (n = 941)							
Age of marriage	0.999	0.037	5.903	1	0.032	2.716	2.526–2.920
BMI	1.192	0.173	9.572	1	0.024	3.293	2.346–4.623
Menstrual regularity	0.654	0.319	6.041	1	0.016	1.924	1.029–3.594
Menstrual flow	0.872	0.312	10.412	1	0.001	2.392	1.298–4.408
Dehong region (n = 813)							
Pelvic inflammatory	1.313	0.157	17.305	1	< 0.001	3.716	2.733–5.057

*CI* confidence interval, *OR* odds ratio

In Kunming region, patients with older marriage age (odds ratio 2.716, P value = 0.032), higher BMI (odds ratio 3.293, P value = 0.024), irregular menstruation (odds ratio 1.924, P value = 0.016), and less menstrual volume (odds ratio 2.392, P value = 0.001) were more likely to be infertile compared to those without. In Dehong region, patients who had pelvic inflammatory disease were almost four times likely to be infertile compared to those without (odds ratio 3.716, P value < 0.001).

### Etiologies of infertility the two regions

Among the 1,754 infertile patients included in the present study, 1,257 (71.66%) had undergone laparoscopy combined with hysteroscopy. Among them, pathologies were found in 1,234 patients, accounting for 98.17% of the total number of infertile patients; 432 cases (33.41%) had pelvic inflammatory disease, 311 cases (24.74%) had hydrosalpinx, 251 cases (19.97%) had an ovarian cyst, 237 cases (18.85%) had hysteromyoma, 165 cases (13.13%) had intrauterine adhesions, and 147 cases (11.69%) had PCOS. Of these 1,257 cases of infertility, 366 (20.05%) possessed two or more of the factors that contribute to infertility. Details are shown in Table 5.

**Table 5:** Comparison of the etiologies of infertility through exploratory operation by laparoscopy combined with hysteroscopy between the two regions

Pathology	Total (n = 1257)	Kunming (n = 651)	Dehong (n = 606)	P value
Pelvic inflammatory	432 (33.41%)	198 (30.34%)	222 (36.63%)	0.037
Slightly adhesion	165 (13.13%)	79 (12.14%)	86 (14.19%)	0.082
Moderate adhesion	155 (12.09%)	73 (11.21%)	79 (13.04%)	0.297
Severe adhesion	103 (8.19%)	46 (7.07%)	57 (9.41%)	0.021
Hydrosalpinx obstruction	311 (24.74%)	141 (21.66%)	170 (28.07%)	0.021
Unilateral obstruction	174 (13.84%)	84 (12.96%)	90 (14.85%)	0.216
Bilateral obstruction	137 (10.90%)	57 (8.76%)	80 (13.20%)	0.042
Endometriosis	140 (11.14%)	71 (10.91%)	69 (11.39%)	0.932
Simple cyst of ovary	93 (7.40%)	51 (7.83%)	42 (6.93%)	0.312
Chocolate cyst of ovary	151 (12.01%)	73 (11.21%)	78 (12.87%)	0.571
Myoma of uterus	237 (18.85%)	122 (18.74%)	115 (18.98%)	0.365
PCOS	147 (11.69%)	84 (12.90%)	63 (10.40%)	0.897
Intrauterine adhesions	165 (13.13%)	111 (17.05%)	54 (8.91%)	0.019
Endometrial polyp	113 (8.99%)	59 (9.06%)	54 (8.91%)	0.953
Pelvic tuberculosis	51 (4.06%)	9 (1.38%)	42 (6.93%)	0.002
Uterine malformation	20 (1.59%)	11 (1.69%)	9 (1.49%)	0.629
Teratoma of ovary	7 (0.56%)	4 (0.614%)	3 (0.50%)	0.891
No complications	23 (1.83%)	14 (2.15%)	9 (1.49%)	0.738

*PCOS* polycystic ovary syndrome

### Etiologies of infertility in patients with primary infertility in the two regions

Among the etiologies of primary infertility in the two regions, ovulation disorder (36.71%) was the main cause in Kunming, while pelvic inflammatory disease (25.59%) was the main cause in Dehong. Additionally, the proportions of tubal factor (P value = 0.034) and pelvic tuberculosis (P value = 0.007) in Dehong were much higher than in Kunming, as can be seen in Table 6.

**Table 6:** Comparison of the etiologies of primary infertility between the two regions

Pathogeny	Kunming (n = 286)	Dehong (n = 379)	P value
Pelvic inflammatory	36 (12.59%)	97 (25.59%)	0.013
Tubal factor	43 (15.03%)	76 (20.05%)	0.034
Ovulation disorders	105 (36.71%)	67 (17.68%)	0.001
Endometriosis	13 (4.55%)	29 (7.65%)	0.892
Ovarian cysts	28 (9.79%)	50 (13.19%)	0.372
Myoma of uterus	27 (9.44%)	47 (12.40%)	0.127
Intrauterine adhesions	10 (3.49%)	19 (5.01%)	0.772
Endometrial polyp	13 (4.57%)	23 (6.07%)	0.561
Pelvic tuberculosis	9 (3.15%)	42 (11.08%)	0.007
Uterine malformation	4 (1.42%)	4 (1.05%)	0.814
Immune factors	35 (12.24%)	40 (10.55%)	0.953
Unknown cause	9 (3.15%)	6 (1.61%)	0.219

### Etiologies of infertility in patients with secondary infertility in the two regions

As shown in Table 7, among the etiologies of secondary infertility, the proportion of pelvic inflammatory disease (28.88% vs. 23.66%, P value = 0.021) and tubal factor (21.61% vs. 16.04%, P value = 0.036) in Dehong was significantly higher than in Kunming, while the proportion of intrauterine adhesions in Kunming was significantly higher than in Dehong (15.42% vs. 8.06%, P value = 0.014).

**Table 7:** Comparison of the etiologies of secondary infertility between the two regions

Pathogeny	Kunming (n = 655)	Dehong (n = 434)	P value
Pelvic inflammatory	155 (23.66%)	125 (28.88%)	0.021
Tubal factor	105 (16.04%)	94 (21.61%)	0.036
Ovulation disorders	80 (12.21%)	54 (12.44%)	0.539
Endometriosis	58 (8.85%)	40 (9.22%)	0.872
Ovarian cysts	64 (9.77%)	46 (10.59%)	0.307
Myoma of uterus	95 (14.66%)	68 (15.67%)	0.105
Intrauterine adhesions	101 (15.42%)	35 (8.06%)	0.014
Endometrial polyp	46 (7.02%)	31 (7.14%)	0.873
Uterine malformation	7 (1.07%)	5 (1.15%)	0.923
Immune factors	70 (10.68%)	46 (10.59%)	0.795
Unknown cause	5 (0.76%)	3 (0.69%)	0.614

## Discussion

The present study revealed that infertile patients in Kunming were mainly affected by such risk factors as late marriage age, being overweight, irregular menstruation, and less menstrual volume, while the infertile patients in Dehong were mainly influenced by the risk factor of previous pelvic inflammatory disease. The differences in etiologies between the two regions were mainly reflected in four factors, including pelvic inflammatory disease, hydrosalpinx, PCOS, and pelvic tuberculosis.

Pelvic inflammation includes the infection and inflammation of the upper genital tract (endometrium, fallopian tubes, ovaries, and pelvic peritoneum) [7–9], which is liable to occur in sexually active females. Becoming sexually active too early, not attending sufficiently to personal hygiene, and sexual activity during menstruation are all factors that make the pelvic cavity prone to invasion by bacteria from the vagina, resulting in pelvic inflammation. The earlier sexual activity is begun, the higher the proportion of pelvic inflammation [10]. The risk factors for pelvic inflammation are having multiple sexual partners, youth, smoking, and using illicit drugs [11]. In recent years, the incidence of pelvic inflammatory disease in China has increased yearly. The results of this clinical investigation showed that the proportion of infertility in patients with pelvic inflammatory disease was about 20–40%. The proportion and duration of occurrences of pelvic inflammatory disease were closely correlated with the proportion of infertility [12].

Pelvic inflammatory disease causes infertility by changing the topography of the pelvic cavity and the internal structure of the fallopian tubes, and ovulation disorders are caused by inflammation around the ovary [13]. In addition, some experts have pointed out that the amount and some characteristics of vaginal secretions in patients with pelvic inflammation might lead to changes in the acid–base environment, which ultimately affects sperm’s activity, thus interfering with fertilization.

In the current study, from the 1,754 infertile patients, 432 had pelvic inflammatory disease, accounting for 24.63% of the total number of patients. The proportion of infertile patients with previous pelvic inflammatory disease in Dehong (15.62%) was higher than in Kunming (5.95%) (P < 0.05). This difference may be correlated with the medical and health conditions, the poor sexual health consciousness, and the younger marriage age in Dehong. Thus, we should note the levels and prevalence of sexual health and hygiene education for childbearing-age couples in Dehong.

Common diseases leading to ovulation disorders in females of childbearing age are PCOS and hyperprolactinemia. Among such females, the incidence of PCOS ranges from 5 to 10%, and the incidence in infertile patients is as high as 30–60% [14]. The phenotype of PCOS is affected by obesity, which also intensifies the metabolic characteristics of PCOS [15]. In the present study, the proportion of ovulation disorders was 17.45%, with a proportion of as high as 36.71% in patients with primary infertility in Kunming. The difference in proportion of ovulation disorders between the two regions may be closely correlated with the differences in BMI, diet structure, work pressure, and endocrine disorders caused by an erratic sleep schedule. Therefore, emphasis should be placed on a healthy lifestyle, moderate exercise regulation, and reasonable diet for the females of childbearing age in Kunming in order to reduce the proportion of ovulation disorders.

Tubal infertility, of which hydrosalpinx is a major cause, accounts for 30–50% of patients with infertility [16]. Inflammation inside the fallopian tube is caused by external infection, salpingitis, or nodular salpingitis (thickening or scarring of the tubal nodules in the isthmus) [17]. Salpingitis may cause an obstruction in and permanent functional injury to the distal end of the fallopian tube. The most common cause of salpingitis is sexually transmitted pelvic inflammation. The percentage of infertility caused by fallopian tube issues in Dehong was much higher than in Kunming, and its main influencing factor was pelvic inflammation. The prevention strategy in this case should thus be similar to that of pelvic inflammation. Actively popularizing reproductive health education and reducing the incidence of sexually transmitted diseases and premarital abortions may have a preventative effect on the reduction of infertility caused by fallopian tube and pelvic diseases.

It has been reported that the incidence of infertility in patients with endometrial adhesions is as high as 90% [18]. Uterine cavity normalcy and sufficient endometrial thickness are essential for a successful pregnancy [19, 20]. Intrauterine adhesions are caused by injury of the basement layer of the endometrium caused by postoperative or inflammatory infection of the uterine cavity, such as abortion and curettage. Scar tissue formed in the uterine cavity can partially or completely occlude the normal cavity, resulting in menstrual abnormalities, infertility, recurrent abortion, and other complications. In the present study, the proportion of intrauterine adhesion in patients with secondary infertility in Kunming (15.42%) was much higher than in Dehong (8.06%) (P < 0.05). In addition, the number of previous pregnancies in patients with secondary infertility in the two regions was different, and this difference was statistically significant. There were significantly more patients with more than two previous pregnancies in Kunming than in Dehong, while the majority of patients with secondary infertility in Dehong were those with only one previous pregnancy (P < 0.05). This might be correlated with the high abortion rate found in infertile patients in Kunming [21]. Thus, according to this difference, we can carry out scientific contraception for females in Kunming, reduce the accidental pregnancy, and weaken the injury of operation to the uterine cavity.

This study has several limitations. First, the cross-sectional nature of the study design might not show the cause and effect relationships between study variables. Second, the samples for this study were taken from the only one teaching hospital of each area. Finally, the sample size of this study was not calculated based on a certain equation.

## Conclusion

In conclusion, there were marked differences in the etiologies and risk factors of infertility between women living in Kunming and Dehong.

## Data Availability

The datasets used and/or analysed during the current study available from the corresponding author on reasonable request.

## Abbreviations

WHO: World Health Organization
BMI: Body mass index
PCOS: Polycystic ovarian syndrome
